# Comparative transcriptome analysis reveals immunoregulation mechanism of lncRNA-mRNA in gill and skin of large yellow croaker (Larimichthys crocea) in response to *C*ryptocaryon irritans infection

**DOI:** 10.1186/s12864-022-08431-w

**Published:** 2022-03-15

**Authors:** Yulin Bai, Mei Wang, Ji Zhao, Huaqiang Bai, Xinyi Zhang, Jiaying Wang, Qiaozhen Ke, Ang Qu, Fei Pu, Weiqiang Zheng, Tao Zhou, Peng Xu

**Affiliations:** 1 State Key Laboratory of Marine Environmental Science, College of Ocean and Earth Sciences, Xiamen University, Xiamen, 361102 China; 2 State Key Laboratory of Large Yellow Croaker Breeding, Ningde Fufa Fisheries Company Limited, Ningde, 352130 China; 3 Fujian Key Laboratory of Genetics and Breeding of Marine Organisms, College of Ocean and Earth Sciences, Xiamen University, Xiamen, 361102 China

**Keywords:** Larimichthys crocea, Cryptocaryon irritans, Comparative transcriptome, Immune response, Immunosuppression, Hub gene

## Abstract

**Background:**

Cryptocaryonosis caused by *Cryptocaryon irritans* is one of the major diseases of large yellow croaker (*Larimichthys crocea*), which lead to massive economic losses annually to the aquaculture industry of *L. crocea*. Although there have been some studies on the pathogenesis for cryptocaryonosis, little is known about the innate defense mechanism of different immune organs of large yellow croaker.

**Results:**

In order to analyze the roles of long non-coding RNAs and genes specifically expressed between immune organs during the infection of *C. irritans*, in this study, by comparing transcriptome data from different tissues of *L. crocea*, we identified tissue-specific transcripts in the gills and skin, including 507 DE lncRNAs and 1592 DEGs identified in the gills, and 110 DE lncRNAs and 1160 DEGs identified in the skin. Furthermore, we constructed transcriptome co-expression profiles of *L. crocea* gill and skin, including 7,503 long noncoding RNAs (lncRNAs) and 23,172 protein-coding genes. Gene Ontology (GO) annotation and Kyoto Encyclopedia of Genes and Genomes (KEGG) pathway analyses showed that the DEGs and the target genes of the DE lncRNAs in the gill were specifically enriched in several pathways related to immune such as HIF-1 signaling pathway. The target genes of DE lncRNAs and DEGs in the skin are specifically enriched in the complement and coagulation cascade pathways. Protein–protein interaction (PPI) network analysis identified 3 hub genes including *NFKBIA*, *TNFAIP3* and *CEBPB*, and 5 important DE lncRNAs including MSTRG.24134.4, MSTRG.3038.5, MSTRG.27019.3, MSTRG.26559.1, and MSTRG.10983.1. The expression patterns of 6 randomly selected differentially expressed immune-related genes were validated using the quantitative real-time PCR method.

**Conclusions:**

In short, our study is helpful to explore the potential interplay between lncRNAs and protein coding genes in different tissues of *L. crocea* post *C. irritans* and the molecular mechanism of pathogenesis for cryptocaryonosis.

**Highlights:**

- Skin and gills are important sources of pro-inflammatory molecules, and their gene expression patterns are tissue-specific after *C. irritans* infection.
- 15 DEGs and 5 DE lncRNAs were identified as hub regulatory elements after *C. irritans* infection
- The HIF-1 signaling pathway and the complement and coagulation cascade pathway may be key tissue-specific regulatory pathways in gills and skin, respectively.

**Supplementary Information:**

The online version contains supplementary material available at 10.1186/s12864-022-08431-w.

## Background

Large yellow croaker (Larimichthys crocea) is one of the most important marine economic fish in China, with a cultured output of 197,000 tons, accounting for about 25% of the global sea bony fish trade volume [1]. Because of its golden color, delicious taste and high protein content, it is very popular among people. However, with the expansion of the aquaculture industry of *L.* crocea, the outbreak of infectious diseases has brought great challenges to the cultivation and breeding of *L*. crocea. Cryptocaryonosis caused by Cryptocaryon irritans is one of the most serious diseases that cause inflammation and death of *L*. crocea [2]. *C.* irritansis is a marine ciliated protozoan parasite that can infect the gills and skin of almost all marine teleost hosts, resulting in the loss of physiological functions of these organs. Although strategies for controlling cryptokaryosis, such as antibiotics, vaccines and metal ions, have been reported, they have only shown weak efficacy under field conditions [3, 4]. Therefore, studying the pathogenesis of cryptogenic diseases is of great significance for improving the disease resistance and breeding level of large yellow croaker.

Parasitic infection is one of the important issues affecting the sustainable development of the marine aquaculture industry (Zhao et al., 2021). In spite of limited pathogen recognition processes, the innate immune system can rapidly discover non-self-recognizing pathogen molecular patterns and send out danger signals to the immune system [5]. At the same time, in teleost, there is still a certain degree of adaptive immunity, including the specific antibody IgT, which binds to the parasite cilia on the surface of the infected fish body to alter parasite behavior and induce an escape reaction [6]. Innate immunity and adaptive immunity work synergistically to maintain the fish's homeostasis [7]. To reduce the impact of diseases on the industry, it is essential to understand the immune mechanisms in fish during pathogenic infections.

Previous studies have shown that fish are able to mount an immune response against parasite infections to inhibit the biological activity of parasites [8–10]. The fish skin is the first line of defense of the immune system and plays various vital functions especially in immunity and defense against invading pathogens and environmental stressors [11, 12]. And the gill is not only involved in gas exchange, but also are major sites for osmoregulation, pH regulation and hormone production [13]. For instance, the *Th2* skew environment represented by the enrichment of pro-inflammatory cytokines in salmon gills and skin can protect fish from parasites and inflammation [14], indicating the importance of differential expression between tissues in innate immunity. The gills and skin are also the primary sites of infection by *C*. irritans displays an extensive cellular response to the pathogen. Recently, the immune functions of fish skin and gill have attracted intensive interests of the research community, and many antimicrobial and bioactive substances have been identified in the tissue mucus [15, 16]. However, there is few transcriptome studies on the tissue-specific immune response of *L. *crocea.

To reduce the impact of parasitic infections on fish industry, in recent years, transcriptome sequencing has been widely used to explore the interactions between host and pathogen. It can not only analyze the structure and expression level of transcripts, but also identify unknown transcriptional isoforms and transcription patterns to accurately analyze valuable issues in life sciences. For instance, the transcriptome analyzed the antibacterial regulation mechanism of the spleen of black carp after infection with Aeromonas hydrophila, and identified the response pathways and key genes related to innate immune [17]. The complex and comprehensive of *Brassica *napus were revealed by using Isoform-sequencing (ISO-seq) technique, and 220,000 alternative splicing events were identified from the genome‐wide full‐length transcripts, which provide a valuable resource for exploring complex transcription patterns, update gene annotation and drive further research on biological gene regulation mechanism [18]. Studies have shown that transcriptome is dominated by one transcript per protein-coding gene, which hinted that not all the transcripts contributing to transcriptome diversity are equally likely to contribute to protein diversity [19]. In addition to protein-coding genes, long noncoding RNAs (lncRNAs) are a class of noncoding RNAs that do not encode proteins. lncRNAs are transcribed from most genomic regions and have important roles in regulating gene expression. More than 1,280 differentially expressed lncRNAs (DE lncRNAs) were identified from ∼2 billion RNA-seq reads in 22 fish families [20]. Using a de novo transcriptome assembly method, Gustavo et al. identified tens of thousands more putative lncRNAs in the Rainbow Trout (Oncorhynchus mykiss) transcriptome [21]. Therefore, transcriptome studies including mRNAs and lncRNAs analysis play key roles in the dissecting of molecular mechanisms of important economic traits.

Although previous studies have sequenced transcriptomes of many *L. crocea* tissues, most of them are single organization expression analysis [22, 23]. In order to gain insights into the transcriptome diversity of *L. crocea* tissues, transcriptome association analysis of multiple tissues still need to be addressed. Fish skin and gills are organs that are directly exposed to external environment, play an important role in protecting the body from pathogen infection [23, 24]. They are also the primary targeting organ and major parasitic site of the *C. irritans* infection, which causes fish hypoxia and bacterial secondary inflammation, and even death [2, 23]. In this study, we reported a comparative transcriptome analysis of gill and skin samples of *L. crocea*. Our main aim was to identify the gills and skin specific transcripts, both protein-coding genes and lncRNAs. We sequenced the whole repertoire of both protein-coding transcripts and lncRNAs from the gills of diseased *L. crocea* at different time points (0 h, 24 h, 48 h, 72 h, and 96 h). Next, we compared the previously reported skin transcriptome data [23] and identified tissue specific protein-coding genes and lncRNAs. It is worth noting that the skin and gill tissue samples are from the same batch of experiments to ensure the comparability of the data. We further analyzed the differentially expressed genes (DEGs), DE lncRNAs and signaling pathway that existed in multiple time points between gills and skin, and considering that they are key members in the immune response to stimulate *C*. irritans infection. The identification of non-tissue-specific hub genes will also support the development of vaccines and environmentally friendly antibacterial agents. Therefore, mRNA and lncRNA comparative transcriptome study will be of great value for the immune mechanism of pathogenic infection, breeding and disease control of *L*. crocea.

## Results

### Overview of sequencing Data and lncRNA Identification

We constructed ribo-depleted libraries of mRNAs and lncRNAs from the *L. crocea* gill infected with *C. irritans* for 0 h, 24 h, 48 h, 72 h, and 96 h and sequenced on Illumina NovaSeq platform. About 43 million pair end high quality reads were collected for each sample respectively (Table S2). The raw RNA-seq data were deposited in Gene Expression Omnibus (GEO) database with accession number GSE174221. To construct a repertoire of *L. crocea* transcripts and compare transcriptome between gill and skin tissues, we also used the skin RNA-seq data reported in previous studies. RNA-seq data from both gill and skin tissues were used for downstream bioinformatic analysis. For each RNA-seq data, we mapped short reads to the *L. crocea* genome by using HISAT2. Based on mapping results, we quantified the expression of known protein-coding genes and lncRNAs using in-house pipeline. Finally, we constructed a stringent set of *L. crocea* RNA transcripts, including 23,172 annotated protein-coding genes and 7,503 lncRNAs.

### Transcriptome variation of L. crocea gill and skin after C. irritans infection

**Fig. 1:**
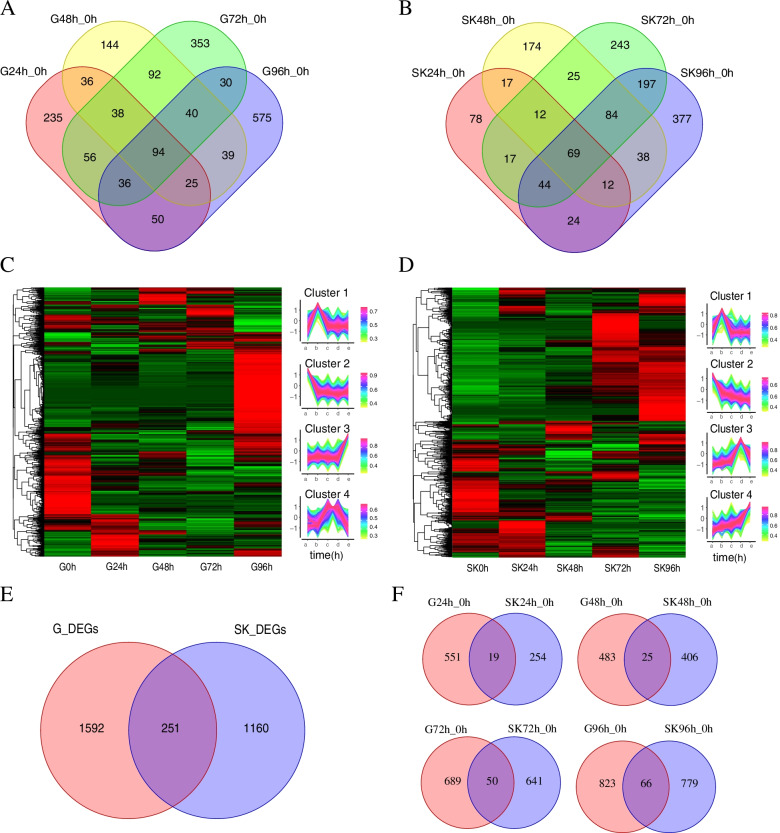
Difference expression analysis of protein coding genes. Venn diagram showing overlapping DEGs among four comparisons in the L. crocea gill (**A**) and skin (**B**) post C. irritans infection; Heatmaps of DEGs in gill (**C**) and skin (**D**) at different infection time points; Venn plot of DEGs in gill and skin at different infection time (**E**, **F**) points

Pairwise comparisons of five analyzed sample groups (0 h, 24 h, 48 h, 72 h, and 96 h) identified a total of 2,706 redundant DEGs in the gill of *L. crocea*, including 1,363 up-regulated DEGs and 1,343 down-regulated DEGs (Figure S1A). Compared with the control group, a set of 570, 508, 739 and 889 DEGs in the gill were identified at 24 h, 48 h, 72 h, and 96 h post *L. crocea* infection, which shows time-dependent DEG increasing (Figure S1A). It is worth noting that after infection with *C. irritans*, the number of up-regulated genes remained stable in the early stage and increased rapidly during 72–96 h, while the number of down-regulated genes increased first and then decreased, reaching a peak at 72 h (Figure S1A). In order to further analyze the interactions among different time points, we constructed a Venn diagram using the DEGs that were differentially expressed in comparisons of G24 h_0 h, G48 h_0 h, G72 h_0 h, and G96 h_0 h, respectively. A total of 536 co-existing DEGs were identified at multiple time points after infection (Fig. 1A). The Heat map showed the different expression patterns of genes in the gill tissue of L. crocea after C. irritans infection, which were divided into 4 clusters (Fig. 1C).

Similarly, a total of 2,240 redundant DEGs were detected in the skin post *L. crocea* infection, including 1,445 up-regulated DEGs and 795 down-regulated DEGs (Figure S1B). Meanwhile, 273, 431, 691 and 845 DEGs in the skin were identified at 24 h, 48 h, 72 h, and 96 h post *L. crocea* infection (Figure S1B). A total of 539 co-existing DEGs were identified at multiple time points after infection (Fig. 1B). Meanwhile, Heat map showed the different expression patterns of genes in the skin tissue of *L. crocea* after *C. irritans* infection, which were divided into 4 clusters (Fig. 1D).

### Tissue-specific analysis of *L. crocea* transcriptome at the gene level

We quantified the expression of protein-coding genes from each RNA-seq data and compared gene expression profiles between the two* L. crocea *tissues. Principal component analysis showed that the samples from the same tissues clustered together, indicating that the protein-coding gene expression values in same tissue showed similar pattern (Figure S3A). Differential expression analysis showed that among the 23,172 protein-coding genes, 3003 non-redundant DEGs have specific expression patterns between gill and skin tissues, including 251 overlapping sequences were also identified both in two tissues, and 1,592 DEGs and 1,160 DEGs were specifically expressed in gill and skin tissues, respectively (Fig. 1E). We also further compared the DEGs of the two tissues at 24 h, 48 h, 72 h and 96 h after infection, and the results showed that a total of 551, 483, 689 and 823 gill tissue-specific DEGs were identified, while the number of skin tissue-specific DEGs at different time points was 254, 406, 641 and 779. (Fig. 1F). We also found that the number of DEGs is increasing with the passage of time post-infection, indicating that their expression patterns were not only tissues-specific but also time-specific (Fig. 1F).

### Function enrichment of differentially expressed mRNAs

To further explore the potential functions of tissue specificity and co-expression of DEGs, we performed GO and KEGG analyses respectively. The non-redundant DEGs were clustered into 4 profiles according to their expression patterns in gills and skin, respectively (Fig. 1C, D). Expression levels of most DEGs in profile 1 (gill and skin) increased continuously until 24 h post-infection, then decreased slightly. KEGG results showed that these genes were mainly enriched in immune pathways including Leukocyte transendothelial migration, IL-17 signaling pathway and HIF-1 signaling pathway in gills, while the main enriched immune pathway in skin was Complement and coagulation cascades pathway. Expression levels of most DEGs in profile 2 (gill and skin) decreased continuously until 24 h post-infection and then remained stable. KEGG results showed that these genes were mainly enriched in immune pathways including Chemokine signaling pathway, NOD-like receptor signaling pathway and Intestinal immune network for IgA production pathway in gills, while the main enriched immune pathway in skin was MAPK signaling pathway. Expression levels of most DEGs in profile 3 (gill) and profile 4 (skin) were consistently up-regulated from 72 to 96 h post-infection. KEGG results showed that these genes were mainly enriched in HIF-1 signaling pathway and metabolic pathway in gills, while the main enriched immune pathways in skin were Complement and coagulation cascades pathway, TNF signaling pathway, IL-17 signaling pathway, NF-kappa B signaling pathway, Toll-like receptor signaling pathway, B cell receptor signaling pathway and C-type lectin receptor signaling pathway. Expression levels of most DEGs in profile 4 (gill) and profile 3 (skin) peaked at 48 or 72 h after infection. The KEGG results showed that these genes were mainly enriched in some metabolic pathways rather than in immune pathways in gills, while the main enriched immune pathway in skin was Antigen processing and presentation pathway.

**Fig. 2:**
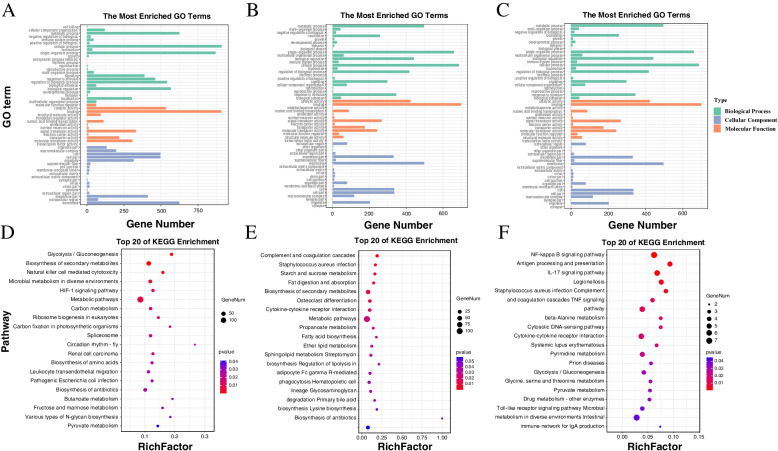
GO enrichment analysis of DEGs in gill (**A**), skin (**B**) and co-exist in gill and skin (**C**) (Lever2 GO terms); Bubble plot of KEGG enrichment analysis of target genes (from left to right were gill-specific DE lncRNAs (**D**), skin-specific DE lncRNAs (**E**), co-exist DE lncRNAs in skin and gill (**F**) [25]

In addition, GO enrichment analysis found that compared with skin, gill tissue-specific DEG is mainly enriched in immune system process, G-protein coupled receptor activity, translation elongation factor activity and cell killing (Fig. 2A, B, Table S3). In addition, GO analysis showed that the overlapped 251 DEGs were classified into 59 sub-categories, of which, 25 in biological process category, 14 in molecular function category, and 20 in cellular component category (Fig. 2C). Based on the above research, we found that multiple immune-related GO terms are enriched, such as the immune system process, immune response, regulation of innate immune response, activation of innate immune response, innate immune response-activating signal transduction, positive regulation of defense response, response to stimulus, MyD88-dependent toll-like receptor signaling pathway. It is worth noting that we have identified multiple immune-related genes, including toll-like receptor 5 (TLR5), ciliary neurotrophic factor (CNTF), C–C motif chemokine 4 (CCL4), tumor necrosis factor alpha-induced protein 3 (TNFAIP3), prostaglandin-endoperoxide synthase 2 (PTGS2).

KEGG enrichment analysis revealed that HIF-1 signaling pathway and Natural killer cell mediated cytotoxicity were significantly enriched in specific DEGs of gill tissue, while complement and coagulation cascades and Toll-like receptor signaling pathway were significantly enriched in specific DEGs of skin tissue (Fig. 2D, E, Table S3). In addition, 251 DEGs co-existed in gill and skin, mainly enriched in NF-kappa B signaling pathway, TNF signaling pathway and Toll-like receptor signaling pathway, Antigen processing and presentation, IL-17 signaling pathway, immune pathways metabolic pathways, and cytokine and cytokine receptor interactions (Fig. 2F, Table S3). Finally, a total of 15 DEGs were identified as important gene set, including growth arrest and DNA-damage-inducible protein (GADD45), NF-kappa-B inhibitor alpha (NFKBIA), tumor necrosis factor alpha-induced protein 3 (TNFAIP3), prostaglandin-endoperoxide synthase 2 (PTGS2), CCL4 TLR5, target of egr1 protein 1 (TOE1), zinc finger protein 710 (Zn710), leptin (LEP), DNA damage-inducible transcript 4 protein (DDIT4), sideroflexin-5 (SFXN5), SPARC-related modular calcium-binding protein 1 (SMOC1), transmembrane protein 181 (TM181), haptoglobin (HPT) and uncharacterized protein LOC104924406 isoform X1 (Table 1).

**Table 1:** Non-tissue-specific hub genes identified from DEGs

Transcript ID	Gene ID	Description
evm.model.LG15.609	*TOE1*	*Target of EGR1 protein 1*
evm.model.LG20.240	*ZN710*	*Zinc finger protein 710*
evm.model.LG20.656	*CCL4*	*C–C motif chemokine 4*
evm.model.LG20.316	*LEP*	*Leptin*
evm.model.LG21.586	*DDIT4*	*DNA damage-inducible transcript 4 protein*
evm.model.LG3.919	*SFXN5*	*Sideroflexin-5*
evm.model.LG3.854.2	*SMOC1*	*SPARC-related modular calcium-binding protein 1*
evm.model.LG12.268.1	*TM181*	*Transmembrane protein 181*
evm.model.LG11.852	*HPT*	*Haptoglobin*
evm.model.LG12.590	*NFKBIA*	*NF-kappa-B inhibitor alpha*
evm.model.LG4.304	*GADD45*	*growth arrest and DNA-damage-inducible protein*
evm.model.LG4.135	*PTGS2*	*prostaglandin-endoperoxide synthase 2*
evm.model.LG21.736	*TNFAIP3*	*tumor necrosis factor, alpha-induced protein 3*
evm.model.LG8.1172	*CEBPB*	*CCAAT/enhancer binding protein (C/EBP), beta*
evm.model.LG12.194	*TLR5*	*toll-like receptor 5*
evm.model.LG6.55	*Unknown*	*PREDICTED: uncharacterized protein LOC104924406 isoform X1*

### Tissue-specific analysis of lncRNAs in *L. crocea* tissues

**Fig. 3:**
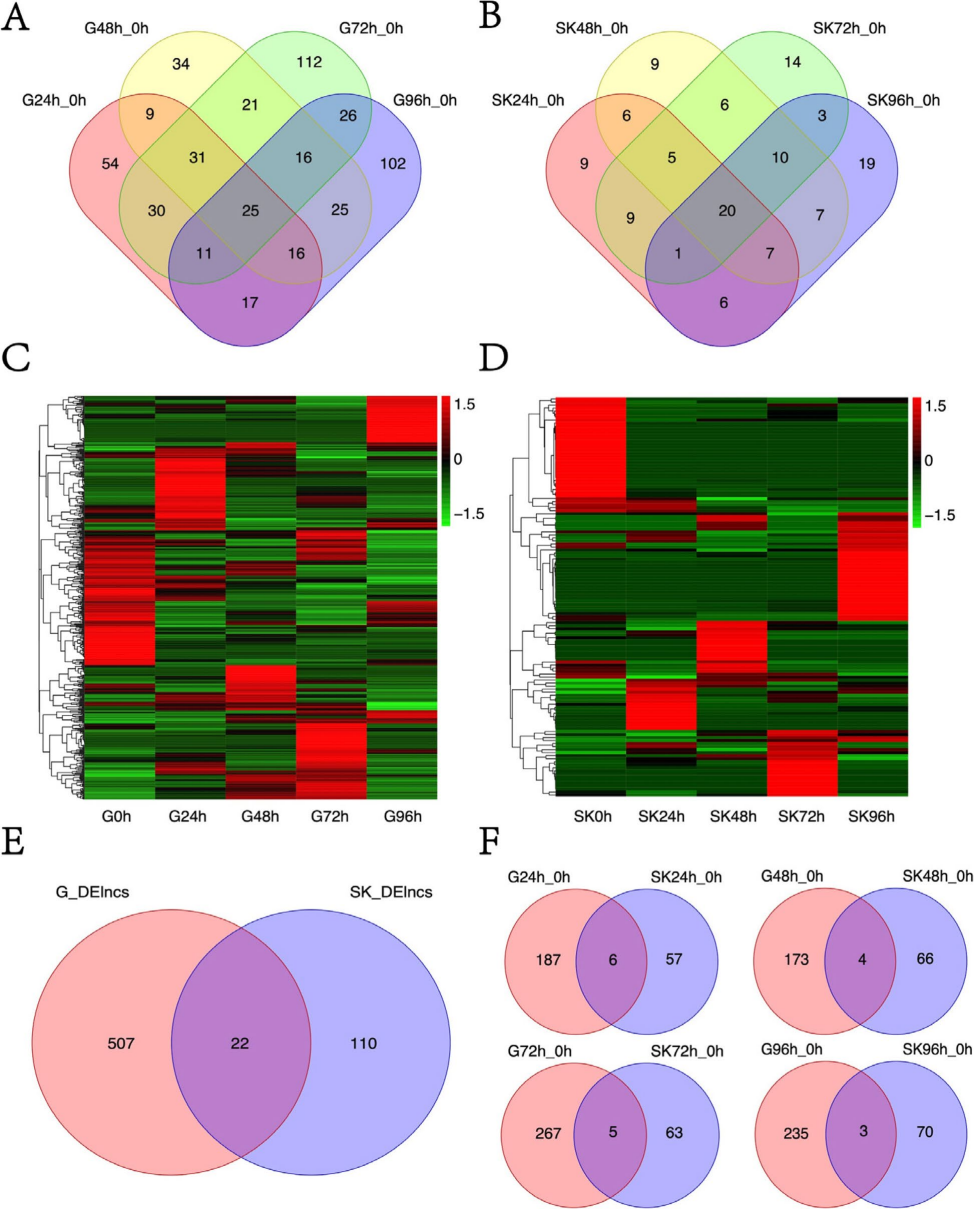
Differential expression of long non-coding RNA. Venn diagram showing overlapping DE lncRNAs among four comparisons in the *L. crocea* gill (**A**) and skin (**B**) post *C. irritans* infection; Heat maps of DE lncRNAs in gill (**C**) and skin (**D**) at different infection time points; Venn plot of DE lncRNAs in gill and skin at different infection time points (**E**, **F**)

In addition to protein-coding genes, lncRNAs are another abundant transcript expressed in *L. crocea* tissues. In comparison to protein-coding genes, lncRNAs had smaller number of exons and shorter transcript length (Figure S2A, B). After *C. irritans* challenge, a total of 880 and 278 DE lncRNAs in the gills and skin, respectively, showed differential expressions at the 4 time points (Figure S1C, D). Principal component analysis of expression values of lncRNAs in these *L. crocea* tissues showed a similar pattern as the protein-coding gene (Figure S3B). To further analyze the interactions among different time points, we respectively constructed a Venn diagram comparing DE lncRNA at different time points of gill and skin infection. A total of 227 overlapping DE lncRNAs in gill and 80 overlapping DE lncRNAs were identified among all the 4 comparisons (Fig. 3A, B). The heatmap of DE-lncRNA both in gill and skin showed each group clustered together indicating differences in the regulatory effects between the two tissues (Fig. 3C, D). Similarly, we calculated the number of tissue-specific DE lncRNAs in *L. crocea* tissues. And the results show that, between gill and skin tissues, there were 507 and 110 tissue-specific DE lncRNAs, respectively (Fig. 3E). In addition, we also the comparison of DE lncRNAs between the gill and skin tissues of *L. crocea* after different infection times of *C. irritans*, indicating that DE lncRNAs are also not only tissue-specific but also time-specific. (Fig. 3F).

### Target gene identification and functional analysis of DE lncRNAs

To identify target genes for DE lncRNAs, we calculated the Pearson’s correlation coefficients between tissue-specific DE lncRNAs and DEGs. A total of 10,433 pairs with high correlation coefficient (|*r*|> 0.99, p-value < 0.001) including 471 unique DE lncRNAs and 1200 unique DEGs were identified. Most of DE lncRNAs were positively correlated with the expression pattern of target genes. The number of target genes ranged from 1 to 230, with an average of 22.

**Fig. 4:**
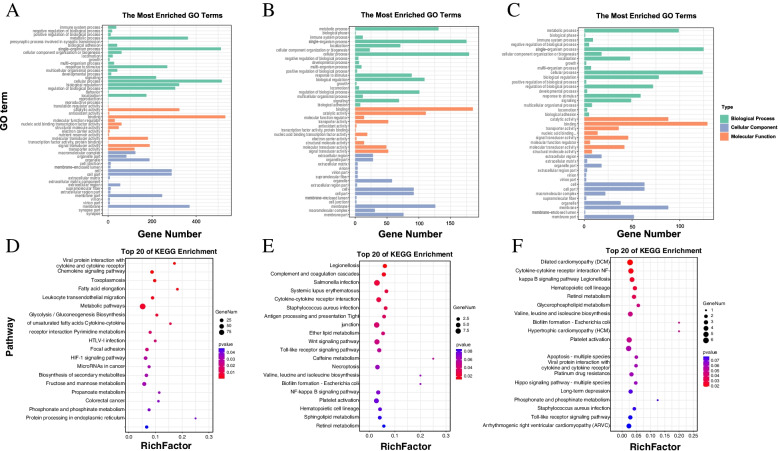
GO enrichment analysis of DE lncRNAs target genes in gill (**A**), skin (**B**) and co-exist in gill and skin (**C**) (Lever2 GO terms); KEGG enrichment analysis of DE lncRNAs target genes in gill (**D**), skin (**E**) and co-exist in gill and skin (**F**) [25]

Similarly, we analyzed the enrichment function of target genes of specific DE lncRNAs in gill and skin tissues (Fig. 4A, B). The significant GO terms of the gill tissue specific DE lncRNAs were mainly associated with “DNA methylation, immune system process”, while the significant GO term of the skin tissue specific DE lncRNAs was related to the regulation of immune response, innate activation of immune response and positive regulation of defense response (Fig. 4A, B). KEGG enrichment results showed that HIF-1 signaling pathway, toxoplasmosis and chemokine signaling pathway were significantly enriched in the target gene of lncRNAs in gill tissue, while complement and coagulation cascades and cytokine-cytokine receptor interaction were significantly enriched in the target gene of DE lncRNAs in skin tissue (Fig. 4D, E). In addition, we enriched the target genes of DE lncRNAs shared in gill and skin, and the results showed that the target genes of DE lncRNAs were Mainly engaged in activation of innate immune response, toll-like receptor signaling pathway, MyD88-dependent toll-like receptor signaling pathway, innate immune response-activating signal transduction, regulation of defense response, positive regulation of defense response, toll-like receptor 5 signaling pathway, regulation of innate immune response (Fig. 4C, F). Moreover, the top 20 enriched pathways of co-exited DE lncRNAs target genes are presented in Table S4.

### PPI and validation of genes related to immune response

**Fig. 5:**
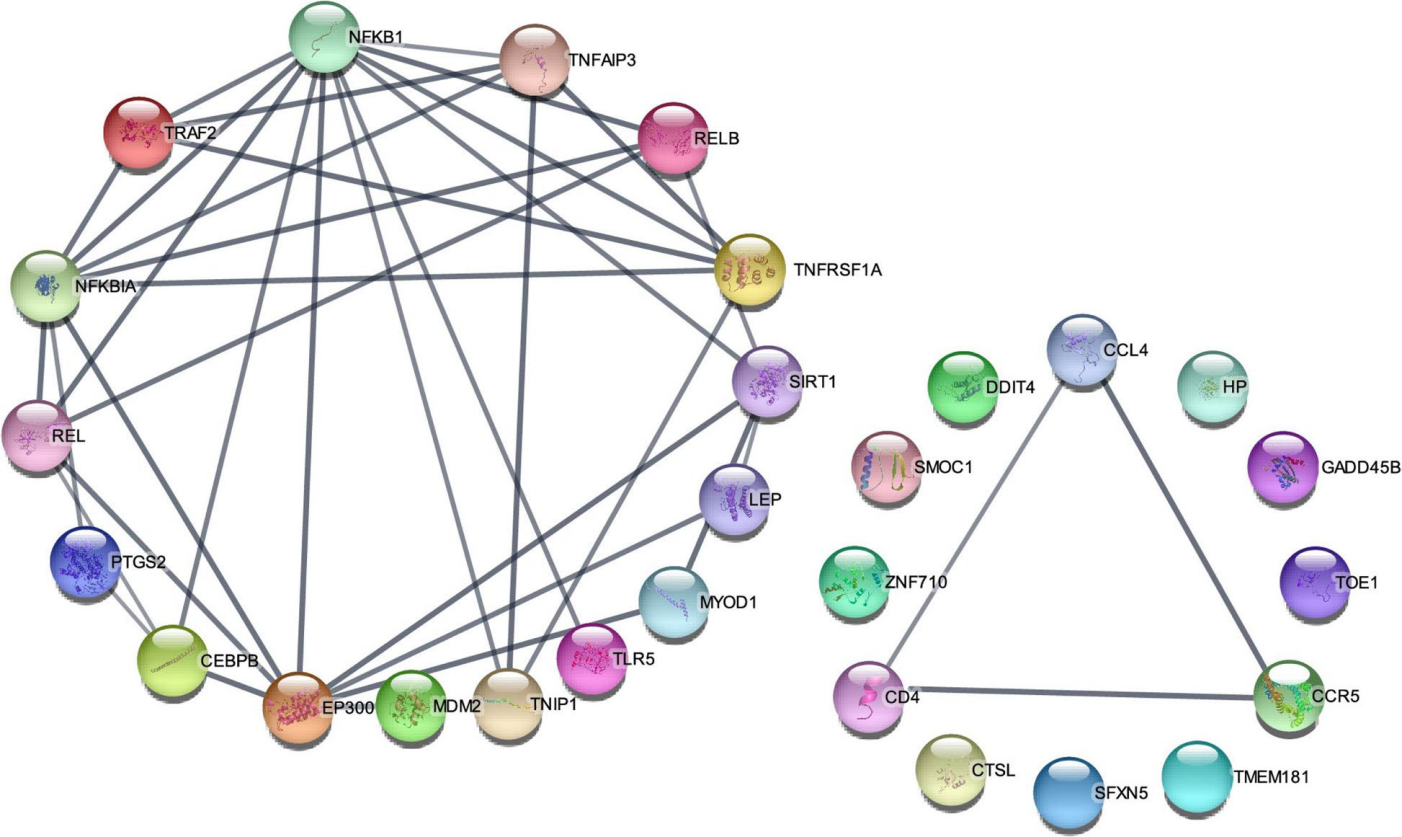
PPI networks of the candidate genes and its interacting protein partners. PPI network constructed using the STRING database shows the immune-related genes and the immune-related genes interacting proteins. The line thickness indicates strength of interaction between any two proteins, and the different colors represent different protein. The colored nodes represent query proteins and first shell of interactors, whereas, white nodes represent second shell of interactors. Max interactors of 1st shell is 6. Max interactors of 2nd shell is 6

To further analyze the relationships among some candidate genes, protein–protein interaction (PPI) network was constructed, including immune-related genes such as *NFKBIA*, *GADD45*, *PTGS2*, *TNFAIP3*, *CEBPB*, *TLR5*, *TOE1*, *Zn710*, *CCL4*, *LEP*, *DDIT4*, *SFXN5*, *SMOC1*, *TM181* and *HPT* (Fig. 5). The network included 28 protein interactions with combined scores that bigger than 0.7 (Max interactors of 1st shell is 6. Max interactors of 2nd shell is 6.). The 3 hub genes including *NFKBIA*, *TNFAIP3* and *CEBPB* with a high level of connectivity were identified.

**Fig. 6:**
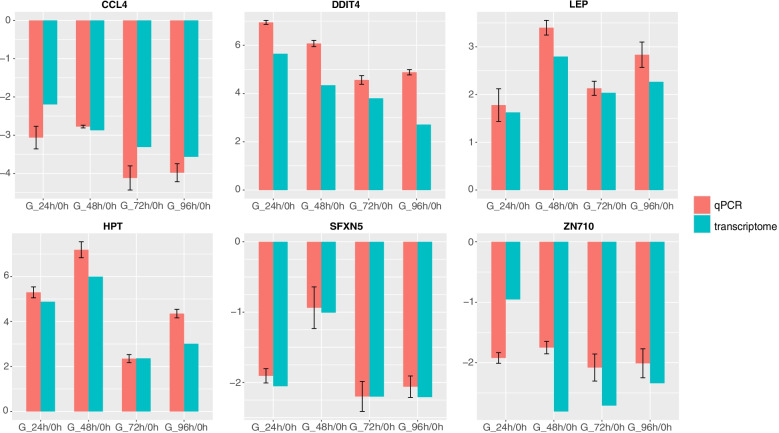
Validation of RNA-seq data by qRT-PCR. Red bar: qRT-PCR; Blue bar: RNA-seq. All expression levels are normalized to the corresponding β-actin mRNA level (qRT-PCR). The data represents the log2 fold change of expression at different time points compared to the control group (G-0 h)

**Fig. 7:**
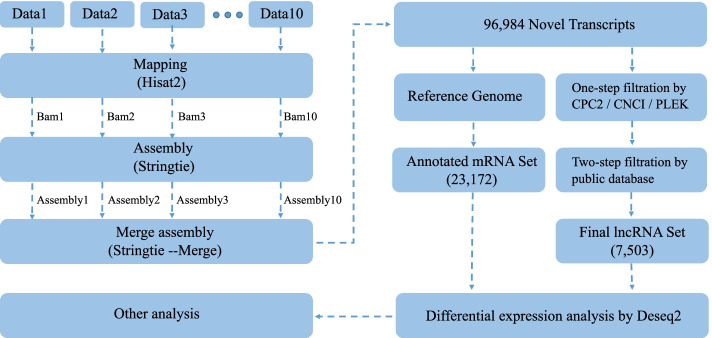
A schematic computational workflow of RNA-seq data in constructing a stringent repertoire of *L. crocea* RNA transcripts

To verify the accuracy of the gene expression profile identified by RNA-seq analysis, the relative mRNA levels of the following six genes (*CCL4*, *DDIT4*, *LEP*, *HPT*, *SFXN5*, *ZN710*) were analyzed by qRT-PCR (Fig. 6). As shown in Fig. 7, the expression pattern of the six genes identified by qRT-PCR was similar to that obtained in the RNA-seq analysis. Therefore, the results of qRT-PCR confirmed the reliability and accuracy of RNA-seq data.

## Discussion

Large yellow croaker is one of the most important marine economic fishes in China [1]. With the increase in market demand and the continuous expansion of large yellow croaker aquaculture, the improvement of large yellow croaker production has received close attention. However, due to its perennial infestation by parasites and pathogens such as *C. irritans*, it has caused huge economic losses. Therefore, in an effort to better understand its pathogenesis, in the present study, we performed transcriptome sequencing on gill tissue samples at 0 h, 24 h, 48 h, 72 h, and 96 h after infection with *C. irritans* in large yellow croaker. And compared the transcriptome data of skin tissue in the same batch of experiments for correlation analysis to reveal the gene tissue-specific expression. A total of 1592 and 1160 tissue-specific DEGs tissue-specific were identified in gills and skin, respectively. We further compared the DEGs of the two tissues at 24 h, 48 h, 72 h and 96 h after infection, and the results showed that a total of 551, 483, 689 and 823 gill tissue-specific DEGs were identified, while the number of skin tissue-specific DEGs at different time points was 254, 406, 641 and 779. These results suggest that the skin and gill tissues of large yellow croaker simultaneously activate the same or different innate immune genes at different time points to defend against *C. irritans* infection. And we also found that the number of DEGs is increasing with the passage of time post-infection. This indicates that with the increase of infection time, more and more immune-related genes of large yellow croaker are involved in the defense mechanism. Similar results were also found in *Sebastiscus marmoratus* and *Epinephelus coioides *[26]; Grouper (*E. coioides*) *CXCR4* is expressed in response to pathogens infection and early stage of development. After stimulation by LPS and necrosis virus, *E. coioides* innate immune genes are differentially expressed in the spleen and eyes [27]. These results indicate that DEGs not only exhibit a high degree of specificity in different tissues, but also have time specificity.

It is noteworthy that the important immune-related genes *CCL4* and *DDIT4*, which are highly associated with *C. irritans* infection, are involved in multiple immune pathways and may play an important role in the regulation of inflammation. As an inflammatory protein, *CCL4* coordinates the immune response to infection or inflammation and promotes the expression of pro-inflammatory cytokines including *TNF—α*, *IL-6* and *IL-1 β* in activated macrophages and fibroblasts during inflammation [28]. Sardar et al. reported that the cooperative induction of *CCL4* is *TLR4-MyD88* dependent and involves NFκB-MAPK-mediated signaling [29]. *DDIT4* is induced by numerous stress stimuli, including pathogen-associated molecular patterns (PAMPs) and hypoxia. *DDIT4* has various biological functions in the oxidative stress and inflammation response process, and mediates immune pathway network to maintain homeostasis [30, 31]. Recent studies have also demonstrated that *DDIT4* is involved in the inflammatory response induced by lipopolysaccharide (LPS) [32]. Though the importance of *CCL4* and *DDIT4* in immune response has been known, their function in *L. crocea* is relatively rarely reported, especially after parasite infection. More basic research is needed to support this result.

In order to understand the interactions of common DEGs in amongst all contrast, GO and KEGG pathway and PPI analyses were performed. GO term analysis of the common DEGs in amongst all contrast was enriched in biological processes (BP), including the immune system process. KEGG enrichment results are consistent with GO annotation results. The common DEGs in amongst all contrast were mainly enriched in immune pathways such as NF-kappa B signaling pathway, IL-17 signaling pathway, and TNF signaling pathway. It has been reported that *IL-17* is elevated in a variety of inflammatory conditions, which can induce the activation of the NF-kappa B pathway and promote the production of pro-inflammatory cytokines such as *IL-1β* and *TNF-α* [33]. Meanwhile, the gene expression profiles of gills and skin showed that DEGs continued to increase after infection, indicating that the fish body has been involved in stimulating the inflammatory response caused by *C. irritans*.

In addition, it has been shown that lncRNA is involved in the immune regulation of teleost fish [34]. For example, of the 5,636 specific lncRNA identified, 3,325 are differentially expressed and most lncRNA associated with innate immune gene during ISA virus (ISAV) infection in the liver of Atlantic salmon [34]. There are 993 DE lncRNAs identified in the liver of Atlantic salmon infected with *Piscirickettsia salmonis* [35]. Similar to coding genes, lncRNAs have high tissue specificity [34, 36]. Consistently, in the present study, a total of 880 and 278 DE lncRNAs in the gills and skin at the 4 time points post *C. irritans* challenge, respectively. In addition, we also the comparison of DE lncRNAs between the gill and skin tissues of *L. crocea* after different infection times of *C. irritans*. The above results indicate that the tissue-specific DE lncRNAs in gills and skin have diversified expression patterns after *C. irritans* infection. The pre-transcriptional regulation of gene expression is quite complicated [37]. As a functional component of post-transcriptional regulation, lncRNA may also act as the main regulator against this stimulus, resulting in a more diverse expression pattern of mRNA and lncRNA [38]. In the process of regulating against exogenous stimulus such as pathogens, lncRNA is induced to have a specific expression pattern compared with mRNA under the same infection state [39]. And the unique expression pattern of some functional lncRNAs may contribute to the accurate and rapid immune response to pathogen stimulation [40]. Our analysis indicated that lncRNAs exhibit specific topological characteristics, and 23 lncRNAs have been identified as being associated with more than 200 target genes, which implies that these lncRNAs may be hubs and control the communication of different network components. In addition, we uncovered five key lncRNAs, MSTRG.24134.4, MSTRG.3038.5, MSTRG.27019.3, MSTRG.26559.1, and MSTRG.10983.1, that were associated with most DEGs including hub genes that have been identified, and may thus be involved in the immune response to stimulating *C. irritans* infection. The above findings reveal the discovery of DE lncRNAs in the *L. crocea* post *C. irritans*, suggesting that lncRNAs might participate in the regulation of host response to parasitic infection, enriching the information of lncRNAs in teleost and providing a resources basis for further studies on the immune function of lncRNAs.

Importantly, the consistency of the enrichment analysis results of DEGs and DE lncRNAs indicates that they may have a certain synergistic relationship in immune regulation and maintenance of body homeostasis. The HIF-1 signaling pathway was identified as the only pathway significantly enriched between DEGs and DE lncRNAs in gill, and play an important role in hypoxia stress, glucose metabolism and autophagy [41, 42]. It is speculated that it may be related to the physical damage caused by stimulation of *C. irritans* to infect gill tissue. It has been reported that mitogen-activated protein kinase (MAPK) pathway can increase HIF-1α expression by inducing extracellular regulated protein kinases (ERK) phosphorylation [43]. In recent years, the MAPK pathway has attracted the attention of researchers as a key immune pathway, and its functions have been reported to be involved in the inflammation regulation and inhibition of apoptosis [44]. It suggests that there may be a MAPK / ERK / HIF-1α cascade immune regulation pathway in gill tissue of *L. crocea*, which plays an important role in the regulation of hypoxia and inflammation caused by *C. irritans* infection. The complement system is a highly complex innate immune pathway, which plays a pivotal role in resisting pathogen infection [45], and the DEGs in skin tissue are significantly enriched in this pathway. The complement system is activated by three pathways: classical, alternative and lectin pathway. And *complement component 3 (C3)* is the key gene of these pathways, which can drive complement effector function to eliminate pathogens and regulate adaptive immunity [46]. We investigated the expression of *C3* in the skin of *L. crocea*, and the expression pattern was up-regulated in 0–48 h, down regulated rapidly in 48–72 h, and gradually returned to normal level in 72–96 h. The up-regulated expression in early stage of infection may be related to the activation of complement system. However, excessive *C3* will produce excessive anaphylactic peptides, which can mediate the aggregation of macrophages and lead to inflammatory response [47]. Therefore, the skin down regulates the level of *C3* transcription to prevent excessive inflammatory responses from damaging self-cells.

To further analyze the relationships among these immune-related DEGs and target genes of DE lncRNAs which were involved in the immune regulation, protein–protein interaction (PPI) network was constructed, including immune-related proteins such as *NFKBIA, GADD45, PTGS2, TNFAIP3, CEBPB, TLR5, TOE1, Zn710, CCL4, LEP, DDIT4, SFXN5, SMOC1, TM181* and *HPT*. The 3 hub genes with a high level of connectivity were identified. The PPI analysis results show that *NFKBIA*, *TNFAIP3* and *CEBPB* were important nodal proteins, which plays an important role in immune regulation.

Interestingly, the results of differential analysis between protein coding genes and lncRNAs showed that the number of transcripts in gills was greater than that in skin at any time point after *C. irritans* infection. It seems that the majority of transcriptional activation occurs within the gills of *L. crocea* compared with the skin. It suggests that the skin had fewer DEGs highlights the localized nature of the host–pathogen interaction. During the experiment, it was observed that the "white spots" on the skin of *L. crocea* were more obviously than the gills after *C. irritans* infection. Studies have shown that once individuals become highly infected with parasite and thus approach the terminal stages of disease, the function of phagocytes is reduced and a down-regulation of many genes occurs [48, 49]. It is speculated that the decrease of DEGs in skin may be related to gene-suppression. In addition, it has also been reported that this gene-suppression may be mediated by the parasite in an attempt to restrict the immune response and thus increase parasite survival [50]. Gene-suppression caused by parasitism is considered to be an important process of interaction and coevolution between parasite and its host [51]. In our study, there may also be some gene-suppression caused by *C. irritans* in the skin tissue of *L. crocea*.

## Conclusions

In summary, the present study first elucidates the immunoregulatory pattern of lncRNA-mRNA in rhubarb on *C. irritans* infection by comparative transcriptome analysis. The results suggest that skin and gills are important sources of pro-inflammatory molecules, and the innate immunity-related genes they mobilize in defense against parasitic infection are not only tissue-specific but also spatiotemporally specific. In particular, three hub protein-coding genes and five hub lncRNAs associated with parasite infection were identified through comparative transcriptome analysis, and their functions involve multiple immune pathways. Importantly, we have identified candidate innate immunity-related genes, including *CCL4* and *DDIT4*, that may play key roles in the anti-inflammatory response during parasite infections. In addition, this study will help to better understand the role of lncRNAs in the immune response of large yellow croaker. Finally, our findings provide an insight into the dynamic characterization of the immune response of large yellow croaker infected with *C. irritans*, This study contributes to a better understanding of the genetic mechanisms underlying the disease resistance traits of large yellow croaker. However, more experimental work is needed to increase our knowledge on the parasite-host model that may help to understand the exact nature of the regulation of immune and other biological processes.

## Materials and methods

### Fish treatment and sample collection

Collection and propagation of the tomonts and theronts of *C. irritans* were conducted using a method described by Dan et al. (2006) [52]. The *L. crocea* (25 ± 10.0 g, n = 200) were cultured at Fufa Aquatic Products Co., Ltd. (Ningde, Fujian) and acclimated for 15 days in a cement tank (26 ± 0.5 °C) before challenge. Before the fish were placed in temporary experimental tank, the water in the tank was fully aerated and exposed to the environment to provide a suitable growth environment for fish. After acclimation, 20 of them were randomly selected and transferred to a temporary tank as control group. The remaining fish averaged into 4 tanks (1 tons) as infected group. Each tank maintains 1000L of water, and only maintains a low water level (200L) during the infection. The fish of infected group were infected with theronts at a dose of 17,000 per fish, and the status of fish was continuously observed for 96 h. During the observation, the fish stopped feeding and the gill tissues of four fish with obvious pathological characteristics were collected from different infection groups at 0 h, 24 h, 48 h, 72 h, and 96 h post-infection. All the sampled fish were anaesthetized by using tricaine methanesulfonate (MS-222; Sigma, St. Louis, MO, USA), and the gill and skin from the same fish were collected simultaneously at each time point, snap frozen in the liquid nitrogen and stored at − 80 °C for subsequent RNA-seq analysis.

### RNA isolation, library preparation and sequencing

Total RNA was isolated using TRIzol reagent (Invitrogen, USA), according to the manufacturer's instructions. Genomic DNA was removed using DNase (New England Biolabs), and RNA purity was assessed using the NanoDrop 2000 (NanoDrop Technologies, USA). The integrity of each RNA sample was detected by agarose gel electrophoresis and had an A260:A280 ratio and A260:A230 ratio and a Qubit 2.0 Fluorometer. The RNA samples were used for the subsequent library construction. Each library was loaded into one lane of the Illumina NovaSeq for 2 × 150 bps pair-end (PE) sequencing.

### Construction of *L. crocea* transcriptome from RNA-seq data

TrimGalore (https://github.com/FelixKrueger/TrimGalore) and FastQC were used to control the quality of sequencing data. At the filtering step, reads that have adaptors, length of reads less than 50, reads quality score less than 20 are regarded as low-quality data and were filtered. The clean reads were aligned to a reference genome of *L. crocea* by Hisat2 (v. 4.8.5). Then, samtools (v. 1.10) was used to filter out the mapping reads which were de-novo assembled separately for each sample by StringTie (v. 2.1.4). Finally, the assembled transcriptomes of each sample were merged to an integrated transcriptome of all tissues by StringTie (v. 2.1.4) using “–merge” option. Gffcompare (v. 0.12.1) was used to compared with known *L. crocea* gene annotation to construct a transcriptome repertoire in *L. crocea* tissues.

The identification of lncRNAs from transcripts follows a strict step-wise pipeline. (i) we used in-house scripts to exclude transcripts smaller than 200 bps; (ii) three different softwares (CPC2, CNCI, and PLEK) were used to estimate the coding potential, and the transcripts identified as coding RNAs by any of the three software were removed; (iii) we used TransDecoder (http://transdecoder.sourceforge.net/) to identify putative open reading frame (ORF) in each transcript, and removed transcripts have putative ORFs longer than 300 bps; (iv) the remaining transcripts were aligned to the open databases including Pfam, Rfam, Uniprot, NR, and miRBase using the program pfamscan, Infernal, and BLAST. All transcripts with alignment E-value < 1e-6 were removed. The set of remaining transcripts were considered as candidate lncRNAs in this study and used for the further analysis. The flow chart of transcriptome construction is shown in Fig. 7.

### Expression quantification and normalization of transcripts in all tissue samples

Based on the Hisat2 alignment BAM file, StringTie and Deseq2 were used to estimate and quantify gene expression separately for each RNA-seq data with default parameters, yielding raw read count and expression abundance for each of the protein-coding genes and lncRNAs across all samples. Gene expression measurements were normalized, we could estimate the fragments per kilobase of exon per million reads mapped (FPKM) using equations:

$$\mathrm{FPKM}=\left(10^6\times{\mathrm n}_{\mathrm f}\right)/\left(\mathrm L\times\mathrm N\right)$$

In these equations, n_f_ is the number of inserts aligned to the gene, L is the sum of the exon lengths of the gene divided by 1000; N is the total effective read counts aligned to the genome.Transcripts expressing differently between any two groups and fulfilling with statistical significance criteria (|log2[foldchange]|≥ 2 and p-value < 0.05) were regarded as DE lncRNAs and DEGs.

### Target gene prediction of DE lncRNAs

The Pearson’s correlation coefficients (r) between each pair of DE lncRNAs and DEGs in *L. crocea* genome were calculated via in-house R scripts (v. 3.6.0). The DEGs with |r|> 0.99 and p-value < 0.001 were considered as the target gene of the paired DE lncRNA.

### Gene expression pattern analysis, functional enrichment of DEGs and PPI network Analyses

Time-course sequencing data analysis (TCseq) was used to assess the expression patterns of non-redundant DEGs over time. By clustering DEGs with similar expression patterns into the same cluster, 4 clusters were obtained in skin and gill tissue respectively.

The functional annotation of genes is based on the reference genome of *L. crocea*. Interprocan (v. 5.0) was used for GO terms annotation. KO terms for each gene are annoted by an online website (KAAS, https://www.genome.jp/tools/kaas/). OmicShare tools (www.omicshare.com/tools) were used to analyze the GO function and KEGG pathway of DEGs, and to explore the corresponding biological functions and related pathways. PPI network construction was performed in STRING (https://string-db.org/), the Search Tool for the Retrieval of Interacting Genes/proteins, referred to the protein interaction result reported in Homo sapiens.

### Quantitative real-time PCR validation

The cDNA was reverse-transcribed with the RNase-free gDNA Eraser kit (TaKaRa, Japan). The five-fold diluted cDNA and SYBR ® Premix Ex Taq™ II (TaKaRa, Japan) were used to perform qRT-PCR with CFX96™ (Bio-Rad, USA). The primers used for the qRT-PCR analyses are listed in Table S1. The PCR cycling conditions used were as follows: 95 °C for 30 s; 40 cycles at 95 °C for 5 s, 60 °C for 30 s and 72 °C for 10 s; and 72 °C for 10 s. The reference gene *β-actin* was used to normalize the expression values. This was followed by a dissociation curve analysis, at 95 °C for 10 s, 60 °C for 1 min and 97 °C for 1 s, to verify the specificity of product amplification. Each experiment group was performed in triplicate.

Data from the qRT-PCR was calculated using the 2 ^−ΔΔCt^ relative quantification method [53]. All data were expressed as the means ± SE. Difference among groups were analyzed by a one-way ANOVA with post-hoc Dunnett's T3 test. *P* < 0.05 was considered to indicate statistical significance.

## Supplementary Information

**Additional File 1**

## Data Availability

The data including mRNA and lncRNA transcript sequences and target gene prediction files have also been deposited at Figshare database (machine-accessible metadata file describing the reported data: 10.6084/m9.figshare.17088794.v1). The RNA-seq raw reads have been deposited in the NCBI Gene Expression Omnibus (GEO) under the GEO accession numbers GPL30112.

## Abbreviations

L. crocea: Large Yellow Croaker
C. irritans: Cryptocaryon irritans
LncRNAs: Long non-coding RNAs
DE lncRNAs: Differentially expressed long non-coding RNAs
DEGs: Differentially expressed genes
NFKBIA: NF-Kappa-B Inhibitor Alpha
GADD45: Growth Arrest and DNA Damage Inducible 45
PTGS2: Prostaglandin-Endoperoxide Synthase 2
CEBPB: CCAAT Enhancer Binding Protein Beta
TLR5: Toll Like Receptor 5
TOE1: Target of EGR1
CCL4: C-C Motif Chemokine Ligand 4
DDIT4: DNA Damage Inducible Transcript 4
CNTF: Ciliary neurotrophic factor
TNFAIP3: Tumor necrosis factor alpha-induced protein 3
Zn710: Zinc finger protein 710
LEP: Leptin
SFXN5: Sideroflexin-5
TM181: Transmembrane protein 181
HPT: Haptoglobin
TNF – α: Tumor Necrosis Factor alpha
IL-6: Interleukin 6
IL-1β: Interleukin 1 Beta
IL-17: Interleukin 17
C3: Complement component 3
ISAV: ISA virus
MAPK: Mitogen-activated protein kinase
ERK: Extracellular regulated protein kinases
qRT PCR: Quantitative real-time PCR
ISO-seq: Isoform-sequencing
RNA-seq: RNA sequencing
PE: Pair-end
ORF: Open reading frame
FPKM: Fragments per kilobase of exon per million reads mapped
KEGG: Kyoto Encyclopedia of Genes and Genomes
GO: Gene ontology
GEO: Gene Expression Omnibus
