# Correction: Comparative transcriptome analysis reveals immunoregulation mechanism of lncRNA-mRNA in gill and skin of large yellow croaker (Larimichthys crocea) in response to Cryptocaryon irritans infection

**DOI:** 10.1186/s12864-022-08615-4

**Published:** 2022-05-16

**Authors:** Yulin Bai, Mei Wang, Ji Zhao, Huaqiang Bai, Xinyi Zhang, Jiaying Wang, Qiaozhen Ke, Ang Qu, Fei Pu, Weiqiang Zheng, Tao Zhou, Peng Xu

**Affiliations:** 1 State Key Laboratory of Marine Environmental Science, College of Ocean and Earth Sciences, Xiamen University, Xiamen, 361102 China; 2 State Key Laboratory of Large Yellow Croaker Breeding, Ningde Fufa Fisheries Company Limited, Ningde, 352130 China; 3 Fujian Key Laboratory of Genetics and Breeding of Marine Organisms, College of Ocean and Earth Sciences, Xiamen University, Xiamen, 361102 China

**Correction: BMC Genomics 23, 206 (2022)**

**https://doi.org/10.1186/s12864-022-08431-w**

Following publication of the original article [1], it was reported that the authorship panel was missing an equal contribution designation for Yuilin Bai and Mei Wang. The original article [1] has been updated to include this.
