# Supplementary material for: Comparative transcriptome analysis reveals immunoregulation mechanism of lncRNA-mRNA in gill and skin of large yellow croaker (Larimichthys crocea) in response to Cryptocaryon irritans infection
Source: BMC Genomics. 2022 Mar 15;23:206. doi: 10.1186/s12864-022-08431-w (PMC8922914; doi:10.1186/s12864-022-08431-w)
Supplement: Supplementary file 1 — Additional File 1 [file 12864_2022_8431_MOESM1_ESM.docx]

**Supplementary Data**

**Additional file 1**

- **Figures**

**Fig. S1 The number of DEGs and DE lncRNA among four pairwise comparisons in gill and skin.** Red spot: log2 (fold change) > 2 and P-value < 0.05; Blue spot: log2 (fold change) < −2 and P-value < 0.05. UP: Up-regulated genes. DOWN: Down-regulated genes.


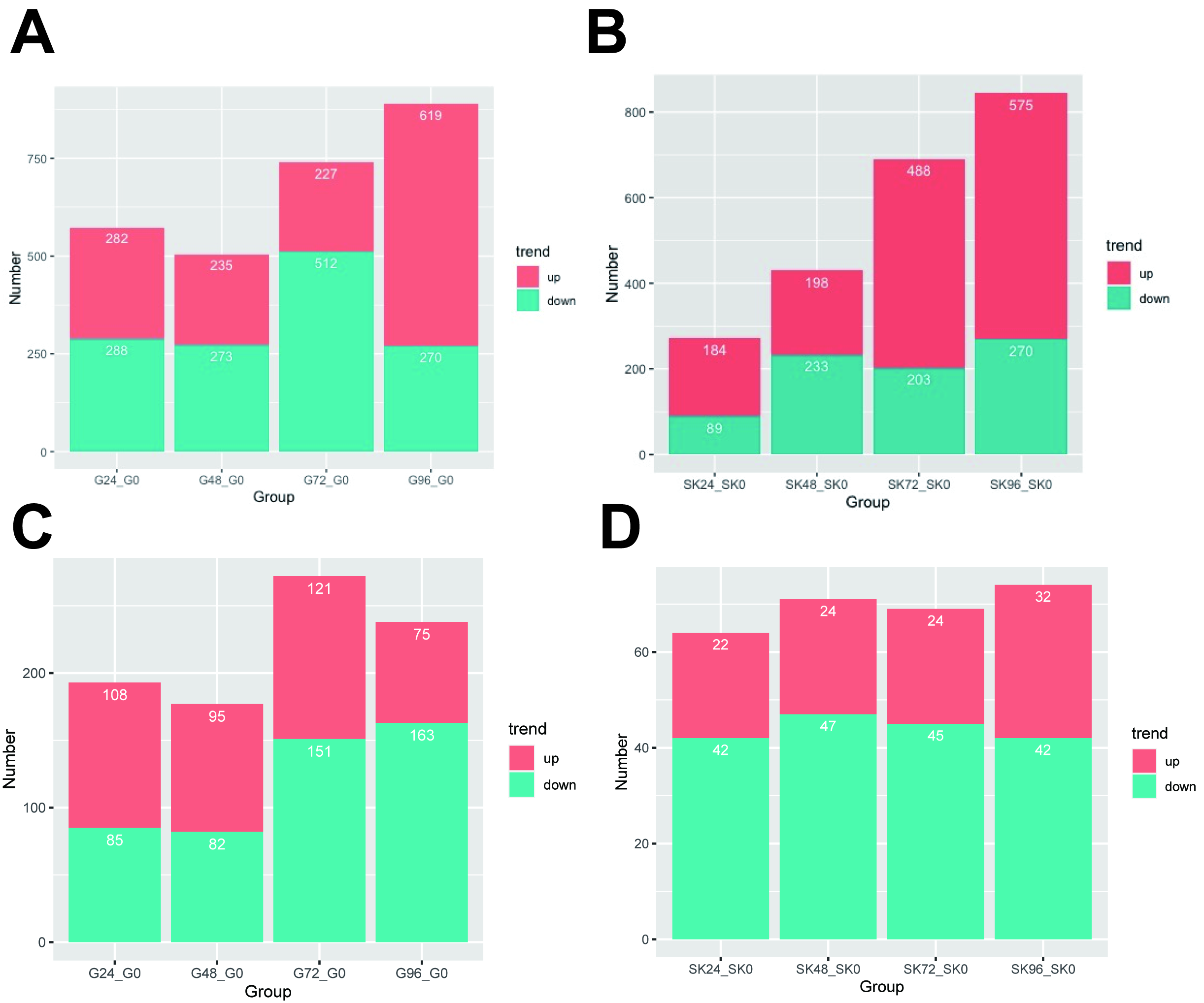


**Fig. S2 Transcript size distribution of mRNA and lncRNA (A); Exon number distribution of mRNA and lncRNA (B).**


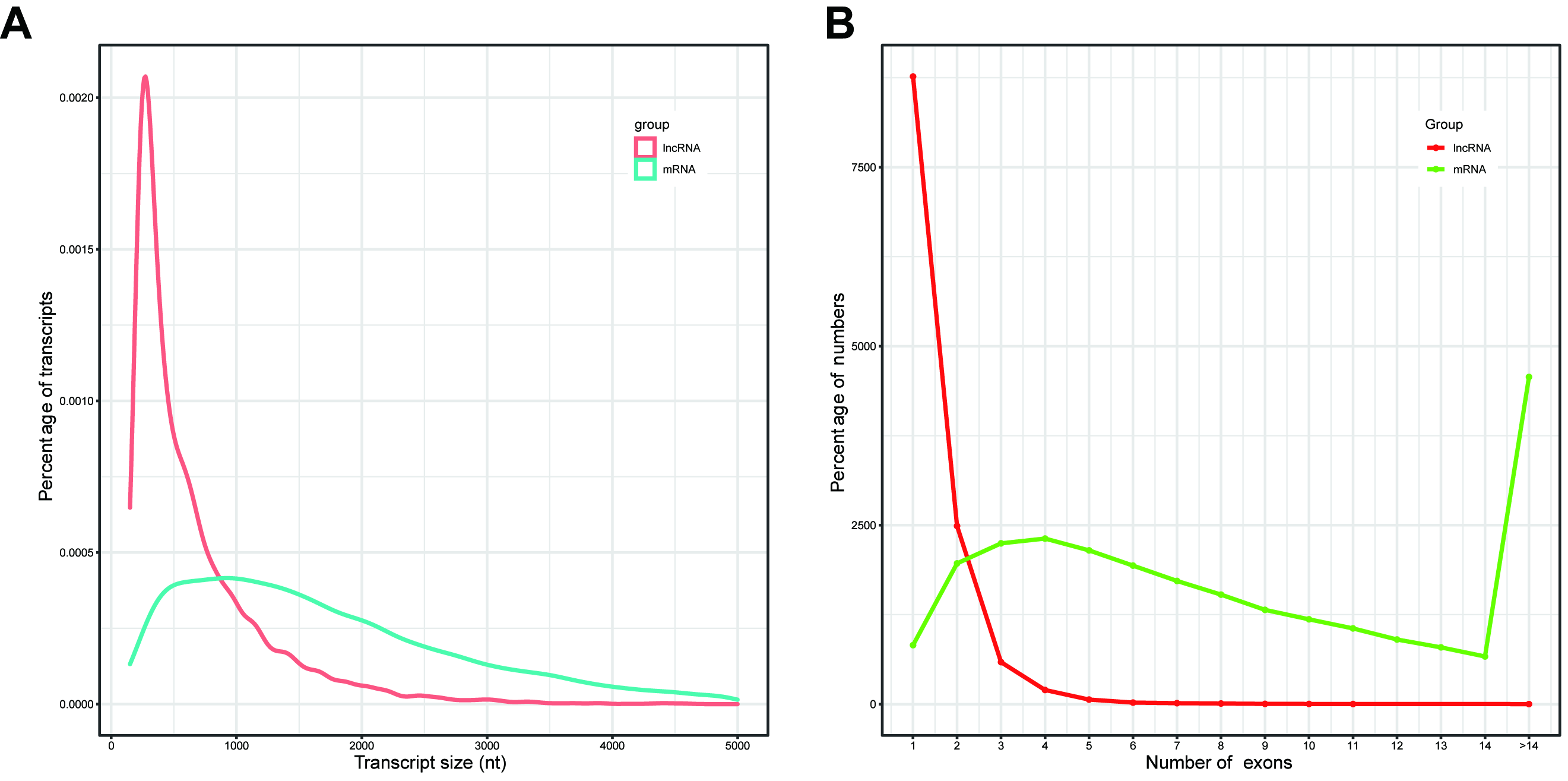


**Fig. S3 Principal component analysis (PCA) based on FPKM among samples (A) PCA analysis of mRNA expression (B) PCA analysis of lncRNAs expression.**


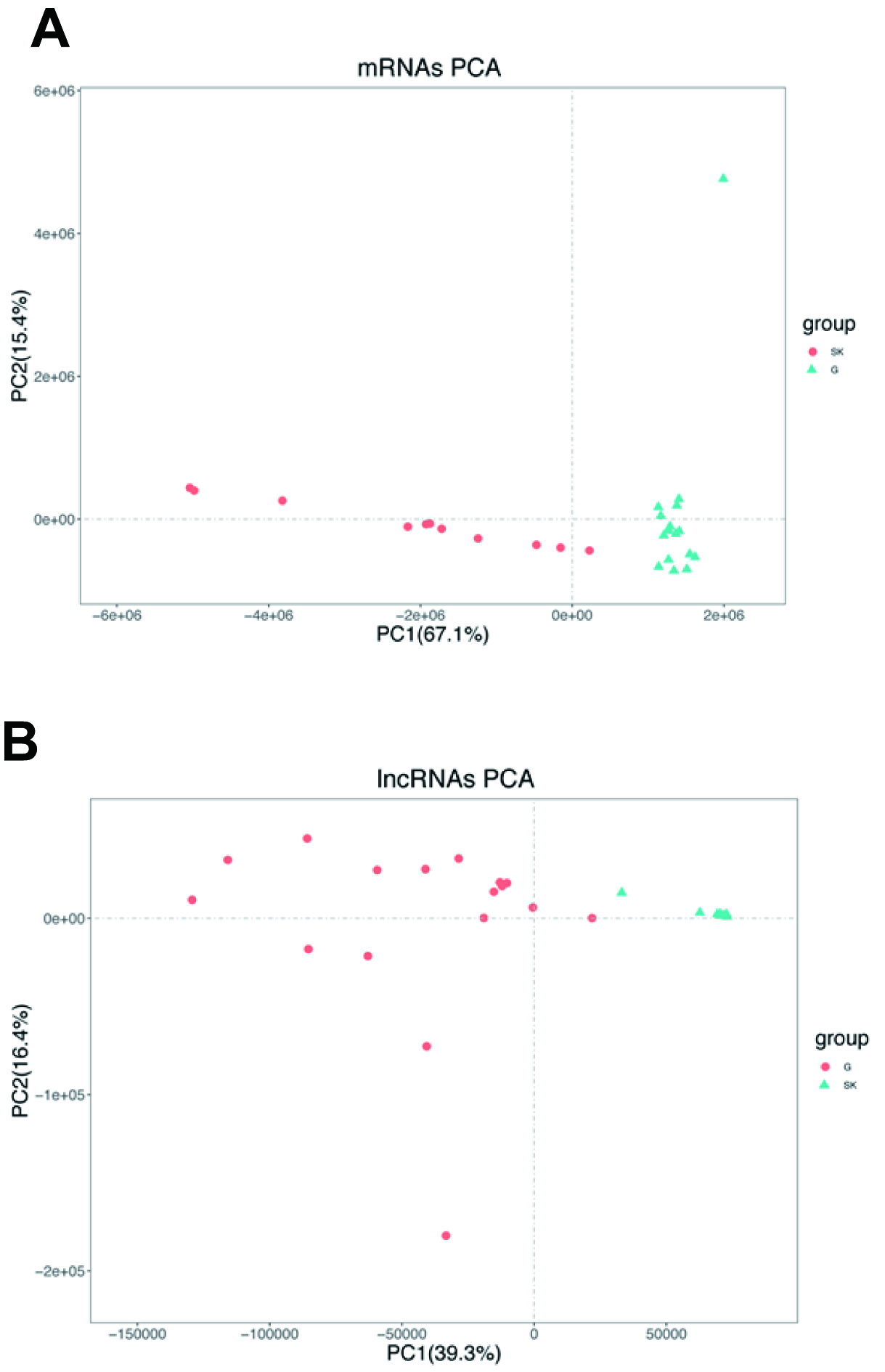


**Tables**

**Table S1** **List of primers for the real-time PCR.**

| **Gene Name** | **Sequence of primers (5'-3')** |
| --- | --- |
| CCL4-Forward | AGCTCACCACCTACCTGGAT |
| CCL4-Reverse | CGTCTGTGACACATGGACGA |
| DDIT4-Forward | GGAGACGTTGGCTACAGAGG |
| DDIT4-Reverse | GAGCCAAGTGGAGCAGTTCT |
| LEP-Forward | CCTGACACTCAGTCCACCTG |
| LEP-Reverse | CCCATCAAGGGTGTCAGAGA |
| HPT-Forward | CATGTTCTGCACCGGACAAC |
| HPT-Reverse | TCCCTGCAGCGTAAATGTCC |
| SFXN5-Forward | AACGTCTGTAACGTGGTGCT |
| SFXN5-Reverse | CGGGTCACGGCTGTTTCTAA |
| ZN710-Forward | AGGGTCTGAAACGCTGGAAG |
| ZN710-Reverse | CAAACGGTGCACTTGTGAGG |

**Table S2 Summary of the Illumina NovaSeq sequencing output and clean reads quality rate.**

| Exported data | | | | | | | |
| --- | --- | --- | --- | --- | --- | --- | --- |
| **sample** | **raw_reads** | **clean_reads** | **clean_bases** | **error_rate** | **Q20** | **Q30** | **GC_pct** |
| G0h1_1 | 41473074 | 40814209 | 12.24G | 0.03 | 97.37 | 92.81 | 45.07 |
| G0h1_5 | 43000144 | 42424048 | 12.73G | 0.03 | 97.65 | 93.33 | 44.88 |
| G0h1_3 | 45667249 | 45113881 | 13.53G | 0.03 | 97.65 | 93.38 | 46.27 |
| G12h1_1 | 42620531 | 41247543 | 12.37G | 0.03 | 97.48 | 93.13 | 45.29 |
| G12h2_3 | 42579343 | 41826510 | 12.55G | 0.03 | 97.48 | 93.05 | 45.51 |
| G12h3_2 | 42469484 | 41482928 | 12.44G | 0.03 | 97.68 | 93.56 | 44.48 |
| G24h1_4 | 42001532 | 40627133 | 12.19G | 0.03 | 97.16 | 92.62 | 45.85 |
| G24h2_3 | 45559059 | 44829777 | 13.45G | 0.03 | 97.4 | 92.93 | 45.48 |
| G24h3_3 | 44325647 | 43665581 | 13.1G | 0.03 | 97.48 | 93.15 | 44.53 |
| G48h1_3 | 43049808 | 41815504 | 12.54G | 0.03 | 97.79 | 93.67 | 44.7 |
| G48h2_2 | 42350723 | 41398787 | 12.42G | 0.03 | 97.36 | 92.73 | 43.5 |
| G48h3_1 | 42203316 | 41125979 | 12.34G | 0.03 | 97.56 | 93.26 | 45.15 |
| G72h1_1 | 43436038 | 42808039 | 12.84G | 0.03 | 97.52 | 93.16 | 46.59 |
| G72h2_6 | 40837912 | 39783561 | 11.94G | 0.03 | 97.52 | 93.28 | 47.64 |
| G72h3_6 | 43104141 | 42406888 | 12.72G | 0.03 | 97.56 | 93.27 | 45.41 |
| G96h1_3 | 43647251 | 42749114 | 12.82G | 0.03 | 97.77 | 93.66 | 48.22 |
| G96h2_4 | 42099464 | 41350678 | 12.41G | 0.03 | 97.69 | 93.44 | 47.48 |
| G96h3_3 | 45324972 | 44504145 | 13.35G | 0.03 | 97.56 | 93.23 | 44.95 |

**Table S3 Enrichment analysis results of DEGs.**

| KEGG enrichment of DEGs in gill (top 20) | | | | | |
| --- | --- | --- | --- | --- | --- |
| KEGG_A_class | KEGG_B_class | Pathway | out (611) | All (8671) | Pvalue |
| Metabolism | Carbohydrate metabolism | Glycolysis / Gluconeogenesis | 18 | 95 | 9.40E-05 |
| Metabolism | Global and overview maps | Biosynthesis of secondary metabolites | 57 | 504 | 0.000211751 |
| Organismal Systems | Immune system | Natural killer cell mediated cytotoxicity | 17 | 106 | 0.001105563 |
| Metabolism | Global and overview maps | Microbial metabolism in diverse environments | 30 | 254 | 0.003515495 |
| Environmental Information Processing | Signal transduction | HIF-1 signaling pathway | 20 | 158 | 0.007474219 |
| Metabolism | Global and overview maps | Metabolic pathways | 135 | 1593 | 0.008942517 |
| Metabolism | Global and overview maps | Carbon metabolism | 21 | 176 | 0.01196042 |
| Genetic Information Processing | Translation | Ribosome biogenesis in eukaryotes | 12 | 83 | 0.01296025 |
| Metabolism | Energy metabolism | Carbon fixation in photosynthetic organisms | 7 | 38 | 0.01546711 |
| Genetic Information Processing | Transcription | Spliceosome | 19 | 160 | 0.01708317 |
| Organismal Systems | Environmental adaptation | Circadian rhythm - fly | 4 | 15 | 0.01782803 |
| Human Diseases | Cancers | Renal cell carcinoma | 15 | 118 | 0.01832281 |
| Metabolism | Global and overview maps | Biosynthesis of amino acids | 15 | 121 | 0.02254199 |
| Organismal Systems | Immune system | Leukocyte transendothelial migration | 20 | 179 | 0.02665689 |
| Human Diseases | Infectious diseases | Pathogenic Escherichia coli infection | 14 | 113 | 0.02707627 |
| Metabolism | Global and overview maps | Biosynthesis of antibiotics | 30 | 297 | 0.02850036 |
| Metabolism | Carbohydrate metabolism | Butanoate metabolism | 5 | 26 | 0.03265905 |
| Metabolism | Carbohydrate metabolism | Fructose and mannose metabolism | 7 | 44 | 0.03267947 |
| Metabolism | Glycan biosynthesis and metabolism | Various types of N-glycan biosynthesis | 5 | 27 | 0.03783947 |
| Metabolism | Carbohydrate metabolism | Pyruvate metabolism | 8 | 56 | 0.04117164 |

KEGG enrichment of DEGs in skin (top 20)

| KEGG_A_class | KEGG_B_class | Pathway | out (474) | All (8671) | Pvalue |
| --- | --- | --- | --- | --- | --- |
| Organismal Systems | Immune system | Complement and coagulation cascades | 13 | 68 | 6.51E-05 |
| Human Diseases | Infectious diseases | Staphylococcus aureus infection | 8 | 47 | 0.003585928 |
| Metabolism | Carbohydrate metabolism | Starch and sucrose metabolism | 8 | 49 | 0.004674538 |
| Organismal Systems | Digestive system | Fat digestion and absorption | 8 | 54 | 0.008500994 |
| Metabolism | Global and overview maps | Biosynthesis of secondary metabolites | 40 | 504 | 0.01055064 |
| Organismal Systems | Development | Osteoclast differentiation | 16 | 158 | 0.01245974 |
| Environmental Information Processing | Signaling molecules and interaction | Cytokine-cytokine receptor interaction | 16 | 162 | 0.01554403 |
| Metabolism | Global and overview maps | Metabolic pathways | 104 | 1593 | 0.02432929 |
| Metabolism | Carbohydrate metabolism | Propanoate metabolism | 6 | 42 | 0.02544288 |
| Metabolism | Lipid metabolism | Fatty acid biosynthesis | 4 | 22 | 0.02943355 |
| Metabolism | Lipid metabolism | Ether lipid metabolism | 7 | 56 | 0.03186295 |
| Metabolism | Lipid metabolism | Sphingolipid metabolism | 8 | 70 | 0.03627722 |
| Metabolism | Biosynthesis of other secondary metabolites | Streptomycin biosynthesis | 3 | 14 | 0.03764635 |
| Organismal Systems | Endocrine system | Regulation of lipolysis in adipocyte | 10 | 97 | 0.03862423 |
| Organismal Systems | Immune system | Fc gamma R-mediated phagocytosis | 14 | 153 | 0.04009121 |
| Organismal Systems | Immune system | Hematopoietic cell lineage | 8 | 72 | 0.04189007 |
| Metabolism | Glycan biosynthesis and metabolism | Glycosaminoglycan degradation | 4 | 25 | 0.04478617 |
| Metabolism | Lipid metabolism | Primary bile acid biosynthesis | 3 | 16 | 0.05344335 |
| Metabolism | Amino acid metabolism | Lysine biosynthesis | 1 | 1 | 0.05466498 |
| Metabolism | Global and overview maps | Biosynthesis of antibiotics | 23 | 297 | 0.05719958 |

KEGG enrichment of DEGs co-exist in gill and skin (top 20)

| KEGG_A_class | KEGG_B_class | Pathway | out (109) | All (8671) | Pvalue |
| --- | --- | --- | --- | --- | --- |
| Environmental Information Processing | Signal transduction | NF-kappa B signaling pathway | 7 | 112 | 0.000501079 |
| Organismal Systems | Immune system | Antigen processing and presentation | 5 | 54 | 0.000554706 |
| Organismal Systems | Immune system | IL-17 signaling pathway | 6 | 88 | 0.00080767 |
| Human Diseases | Infectious diseases | Legionellosis | 5 | 66 | 0.00139136 |
| Human Diseases | Infectious diseases | Staphylococcus aureus infection | 4 | 47 | 0.002781308 |
| Organismal Systems | Immune system | Complement and coagulation cascades | 4 | 68 | 0.01038853 |
| Environmental Information Processing | Signal transduction | TNF signaling pathway | 6 | 154 | 0.01287217 |
| Metabolism | Metabolism of other amino acids | beta-Alanine metabolism | 3 | 40 | 0.01362476 |
| Organismal Systems | Immune system | Cytosolic DNA-sensing pathway | 3 | 40 | 0.01362476 |
| Environmental Information Processing | Signaling molecules and interaction | Cytokine-cytokine receptor interaction | 6 | 162 | 0.01618161 |
| Human Diseases | Immune diseases | Systemic lupus erythematosus | 3 | 45 | 0.01870601 |
| Metabolism | Nucleotide metabolism | Pyrimidine metabolism | 5 | 128 | 0.02237513 |
| Human Diseases | Neurodegenerative diseases | Prion diseases | 3 | 54 | 0.03016047 |
| Metabolism | Carbohydrate metabolism | Glycolysis / Gluconeogenesis | 4 | 95 | 0.03144532 |
| Metabolism | Amino acid metabolism | Glycine, serine and threonine metabolism | 3 | 55 | 0.03161633 |
| Metabolism | Carbohydrate metabolism | Pyruvate metabolism | 3 | 56 | 0.03310854 |
| Metabolism | Xenobiotics biodegradation and metabolism | Drug metabolism - other enzymes | 3 | 57 | 0.03463698 |
| Organismal Systems | Immune system | Toll-like receptor signaling pathway | 4 | 102 | 0.03930036 |
| Metabolism | Global and overview maps | Microbial metabolism in diverse environments | 7 | 254 | 0.04064244 |
| Organismal Systems | Immune system | Intestinal immune network for IgA production | 2 | 27 | 0.04481303 |

GO enrichment of DEGs in gill (top 20)

| GO ID | Description | out (1348) | All (19473) | pvalue |
| --- | --- | --- | --- | --- |
| GO:0008009 | chemokine activity | 10 | 48 | 0.001417103 |
| GO:0042379 | chemokine receptor binding | 10 | 48 | 0.001417103 |
| GO:0004571 | mannosyl-oligosaccharide 1,2-alpha-mannosidase activity | 5 | 17 | 0.004834758 |
| GO:0015924 | mannosyl-oligosaccharide mannosidase activity | 5 | 17 | 0.004834758 |
| GO:0004559 | alpha-mannosidase activity | 6 | 25 | 0.006151658 |
| GO:0001664 | G-protein coupled receptor binding | 13 | 88 | 0.007358952 |
| GO:0015923 | mannosidase activity | 6 | 26 | 0.007537274 |
| GO:0003779 | actin binding | 19 | 153 | 0.009485107 |
| GO:0008092 | cytoskeletal protein binding | 39 | 384 | 0.010520604 |
| GO:0005544 | calcium-dependent phospholipid binding | 13 | 93 | 0.011580478 |
| GO:0019829 | cation-transporting ATPase activity | 20 | 168 | 0.012369126 |
| GO:0042625 | ATPase coupled ion transmembrane transporter activity | 20 | 168 | 0.012369126 |
| GO:0004859 | phospholipase inhibitor activity | 5 | 21 | 0.012622448 |
| GO:0055102 | lipase inhibitor activity | 5 | 21 | 0.012622448 |
| GO:0004809 | tRNA (guanine-N2-)-methyltransferase activity | 2 | 3 | 0.013703917 |
| GO:0008762 | UDP-N-acetylmuramate dehydrogenase activity | 2 | 3 | 0.013703917 |
| GO:0005539 | glycosaminoglycan binding | 6 | 30 | 0.015354147 |
| GO:0016903 | oxidoreductase activity, acting on the aldehyde or oxo group of donors | 6 | 32 | 0.020838601 |
| GO:0005125 | cytokine activity | 10 | 70 | 0.021778544 |
| GO:0005540 | hyaluronic acid binding | 5 | 24 | 0.022203759 |

GO enrichment of DEGs in skin (top 20)

| GO ID | Description | out (1007) | All (19473) | pvalue |
| --- | --- | --- | --- | --- |
| GO:0003810 | protein-glutamine gamma-glutamyltransferase activity | 4 | 9 | 0.000726503 |
| GO:0004934 | mating-type alpha-factor pheromone receptor activity | 5 | 19 | 0.002318067 |
| GO:0003743 | translation initiation factor activity | 5 | 20 | 0.00296063 |
| GO:0003824 | catalytic activity | 423 | 7386 | 0.003564357 |
| GO:0004198 | calcium-dependent cysteine-type endopeptidase activity | 6 | 30 | 0.003842772 |
| GO:0043169 | cation binding | 205 | 3362 | 0.00496404 |
| GO:0046872 | metal ion binding | 203 | 3340 | 0.005977182 |
| GO:0035091 | phosphatidylinositol binding | 8 | 57 | 0.008653114 |
| GO:0008308 | voltage-gated anion channel activity | 10 | 81 | 0.008785964 |
| GO:0004197 | cysteine-type endopeptidase activity | 9 | 70 | 0.009754257 |
| GO:0004415 | hyalurononglucosaminidase activity | 4 | 17 | 0.009851956 |
| GO:0015929 | hexosaminidase activity | 4 | 17 | 0.009851956 |
| GO:0017016 | Ras GTPase binding | 7 | 47 | 0.00997077 |
| GO:0043167 | ion binding | 366 | 6412 | 0.010141428 |
| GO:0005488 | binding | 699 | 12862 | 0.010854418 |
| GO:0031267 | small GTPase binding | 7 | 48 | 0.011165921 |
| GO:0051020 | GTPase binding | 7 | 48 | 0.011165921 |
| GO:0008140 | cAMP response element binding protein binding | 2 | 4 | 0.014948204 |
| GO:0004713 | protein tyrosine kinase activity | 45 | 623 | 0.015032057 |
| GO:0036318 | peptide pheromone receptor activity | 5 | 29 | 0.015442897 |

GO enrichment of DEGs co-exist in gill and skin (top 20)

| GO ID | Description | out (223) | All (19473) | pvalue |
| --- | --- | --- | --- | --- |
| GO:0005125 | cytokine activity | 9 | 70 | 1.02E-07 |
| GO:0008009 | chemokine activity | 7 | 48 | 1.16E-06 |
| GO:0042379 | chemokine receptor binding | 7 | 48 | 1.16E-06 |
| GO:0001664 | G-protein coupled receptor binding | 7 | 88 | 6.80E-05 |
| GO:0005507 | copper ion binding | 3 | 31 | 0.005257903 |
| GO:0005126 | cytokine receptor binding | 10 | 346 | 0.006746605 |
| GO:0046872 | metal ion binding | 53 | 3340 | 0.006993235 |
| GO:0043169 | cation binding | 53 | 3362 | 0.007963848 |
| GO:0004867 | serine-type endopeptidase inhibitor activity | 4 | 71 | 0.008941871 |
| GO:0004857 | enzyme inhibitor activity | 6 | 161 | 0.010701462 |
| GO:0016641 | oxidoreductase activity, acting on the CH-NH2 group of donors, oxygen as acceptor | 2 | 18 | 0.01770487 |
| GO:0046914 | transition metal ion binding | 28 | 1616 | 0.018174924 |
| GO:0016638 | oxidoreductase activity, acting on the CH-NH2 group of donors | 2 | 19 | 0.019640285 |
| GO:0004329 | formate-tetrahydrofolate ligase activity | 1 | 2 | 0.022772946 |
| GO:0005415 | nucleoside:sodium symporter activity | 1 | 2 | 0.022772946 |
| GO:0004540 | ribonuclease activity | 2 | 21 | 0.023761894 |
| GO:0004866 | endopeptidase inhibitor activity | 4 | 98 | 0.026233299 |
| GO:0061135 | endopeptidase regulator activity | 4 | 98 | 0.026233299 |
| GO:0030234 | enzyme regulator activity | 12 | 556 | 0.026951135 |
| GO:0003735 | structural constituent of ribosome | 7 | 255 | 0.027901145 |

**Table S4 Enrichment analysis results of the target gene of lncRNAs.**

KEGG enrichment of target genes of DElncRNAs in gill (top 20)

| KEGG_A_class | KEGG_B_class | Pathway | out (373) | All (8976) | Pvalue |
| --- | --- | --- | --- | --- | --- |
| Environmental Information Processing | Signaling molecules and interaction | Viral protein interaction with cytokine and cytokine receptor | 7 | 41 | 0.001338363 |
| Organismal Systems | Immune system | Chemokine signaling pathway | 20 | 229 | 0.001341295 |
| Human Diseases | Infectious diseases | Toxoplasmosis | 15 | 155 | 0.001943284 |
| Metabolism | Lipid metabolism | Fatty acid elongation | 6 | 33 | 0.002111614 |
| Organismal Systems | Immune system | Leukocyte transendothelial migration | 16 | 179 | 0.003141569 |
| Metabolism | Global and overview maps | Metabolic pathways | 97 | 1848 | 0.005885277 |
| Metabolism | Carbohydrate metabolism | Glycolysis / Gluconeogenesis | 10 | 95 | 0.005917179 |
| Metabolism | Lipid metabolism | Biosynthesis of unsaturated fatty acids | 5 | 32 | 0.009624394 |
| Environmental Information Processing | Signaling molecules and interaction | Cytokine-cytokine receptor interaction | 13 | 162 | 0.01730419 |
| Metabolism | Nucleotide metabolism | Pyrimidine metabolism | 8 | 81 | 0.01887872 |
| Human Diseases | Infectious diseases | HTLV-I infection | 20 | 294 | 0.02056797 |
| Cellular Processes | Cellular community - eukaryotes | Focal adhesion | 23 | 364 | 0.02944688 |
| Environmental Information Processing | Signal transduction | HIF-1 signaling pathway | 12 | 158 | 0.03162398 |
| Human Diseases | Cancers | MicroRNAs in cancer | 19 | 289 | 0.0320926 |
| Metabolism | Global and overview maps | Biosynthesis of secondary metabolites | 32 | 550 | 0.03280877 |
| Metabolism | Carbohydrate metabolism | Fructose and mannose metabolism | 5 | 44 | 0.03468887 |
| Metabolism | Carbohydrate metabolism | Propanoate metabolism | 5 | 45 | 0.03774667 |
| Human Diseases | Cancers | Colorectal cancer | 11 | 145 | 0.03888566 |
| Metabolism | Metabolism of other amino acids | Phosphonate and phosphinate metabolism | 2 | 8 | 0.04084231 |
| Genetic Information Processing | Folding, sorting and degradation | Protein processing in endoplasmic reticulum | 15 | 224 | 0.04640238 |

KEGG enrichment of target genes of DElncRNAs in skin (top 20)

| KEGG_A_class | KEGG_B_class | Pathway | out (125) | All (8976) | Pvalue |
| --- | --- | --- | --- | --- | --- |
| Human Diseases | Infectious diseases | Legionellosis | 4 | 66 | 0.01329551 |
| Organismal Systems | Immune system | Complement and coagulation cascades | 4 | 70 | 0.0162143 |
| Human Diseases | Infectious diseases | Salmonella infection | 9 | 301 | 0.02445061 |
| Human Diseases | Immune diseases | Systemic lupus erythematosus | 3 | 45 | 0.02446574 |
| Environmental Information Processing | Signaling molecules and interaction | Cytokine-cytokine receptor interaction | 6 | 162 | 0.02545378 |
| Human Diseases | Infectious diseases | Staphylococcus aureus infection | 3 | 47 | 0.02740517 |
| Organismal Systems | Immune system | Antigen processing and presentation | 3 | 52 | 0.03553936 |
| Cellular Processes | Cellular community - eukaryotes | Tight junction | 8 | 279 | 0.04078622 |
| Metabolism | Lipid metabolism | Ether lipid metabolism | 3 | 56 | 0.04284375 |
| Environmental Information Processing | Signal transduction | Wnt signaling pathway | 7 | 236 | 0.04650477 |
| Organismal Systems | Immune system | Toll-like receptor signaling pathway | 4 | 102 | 0.05374051 |
| Metabolism | Biosynthesis of other secondary metabolites | Caffeine metabolism | 1 | 4 | 0.05456019 |
| Cellular Processes | Cell growth and death | Necroptosis | 5 | 156 | 0.06650288 |
| Metabolism | Amino acid metabolism | Valine, leucine and isoleucine biosynthesis | 1 | 5 | 0.06773228 |
| Cellular Processes | Cellular community - prokaryotes | Biofilm formation - Escherichia coli | 1 | 5 | 0.06773228 |
| Environmental Information Processing | Signal transduction | NF-kappa B signaling pathway | 4 | 113 | 0.07265095 |
| Organismal Systems | Immune system | Platelet activation | 6 | 213 | 0.0767371 |
| Organismal Systems | Immune system | Hematopoietic cell lineage | 3 | 72 | 0.07874853 |
| Metabolism | Lipid metabolism | Sphingolipid metabolism | 3 | 74 | 0.08393627 |
| Metabolism | Metabolism of cofactors and vitamins | Retinol metabolism | 2 | 35 | 0.08501375 |

KEGG enrichment of target genes of DElncRNAs co-exist in gill and skin (top 20)

| KEGG_A_class | KEGG_B_class | Pathway | out (88) | All (8976) | Pvalue |
| --- | --- | --- | --- | --- | --- |
| Human Diseases | Cardiovascular diseases | Dilated cardiomyopathy (DCM) | 6 | 217 | 0.0195297 |
| Environmental Information Processing | Signaling molecules and interaction | Cytokine-cytokine receptor interaction | 5 | 162 | 0.02123704 |
| Environmental Information Processing | Signal transduction | NF-kappa B signaling pathway | 4 | 113 | 0.02474675 |
| Human Diseases | Infectious diseases | Legionellosis | 3 | 66 | 0.02674706 |
| Organismal Systems | Immune system | Hematopoietic cell lineage | 3 | 72 | 0.03343955 |
| Metabolism | Metabolism of cofactors and vitamins | Retinol metabolism | 2 | 35 | 0.0458804 |
| Metabolism | Lipid metabolism | Glycerophospholipid metabolism | 4 | 139 | 0.04736813 |
| Metabolism | Amino acid metabolism | Valine, leucine and isoleucine biosynthesis | 1 | 5 | 0.04807832 |
| Cellular Processes | Cellular community - prokaryotes | Biofilm formation - Escherichia coli | 1 | 5 | 0.04807832 |
| Human Diseases | Cardiovascular diseases | Hypertrophic cardiomyopathy (HCM) | 5 | 204 | 0.04970155 |
| Organismal Systems | Immune system | Platelet activation | 5 | 213 | 0.05779144 |
| Cellular Processes | Cell growth and death | Apoptosis - multiple species | 2 | 40 | 0.05829753 |
| Environmental Information Processing | Signaling molecules and interaction | Viral protein interaction with cytokine and cytokine receptor | 2 | 41 | 0.06090667 |
| Human Diseases | Drug resistance | Platinum drug resistance | 3 | 92 | 0.0613465 |
| Environmental Information Processing | Signal transduction | Hippo signaling pathway - multiple species | 2 | 42 | 0.0635553 |
| Organismal Systems | Nervous system | Long-term depression | 3 | 100 | 0.07478276 |
| Metabolism | Metabolism of other amino acids | Phosphonate and phosphinate metabolism | 1 | 8 | 0.07582079 |
| Human Diseases | Infectious diseases | Staphylococcus aureus infection | 2 | 47 | 0.07735614 |
| Organismal Systems | Immune system | Toll-like receptor signaling pathway | 3 | 102 | 0.07833073 |
| Human Diseases | Cardiovascular diseases | Arrhythmogenic right ventricular cardiomyopathy (ARVC) | 4 | 165 | 0.07850342 |

GO enrichment of target genes of DElncRNAs in gill (top 20)

| GO ID | Description | out (801) | All (19473) | pvalue |
| --- | --- | --- | --- | --- |
| GO:0008009 | chemokine activity | 11 | 48 | 2.98E-06 |
| GO:0042379 | chemokine receptor binding | 11 | 48 | 2.98E-06 |
| GO:0005125 | cytokine activity | 12 | 70 | 2.56E-05 |
| GO:0001664 | G-protein coupled receptor binding | 13 | 88 | 6.07E-05 |
| GO:0051537 | 2 iron, 2 sulfur cluster binding | 3 | 10 | 0.006697444 |
| GO:0019825 | oxygen binding | 6 | 42 | 0.007043336 |
| GO:0005126 | cytokine receptor binding | 24 | 346 | 0.009075921 |
| GO:0004177 | aminopeptidase activity | 5 | 34 | 0.012023629 |
| GO:0003796 | lysozyme activity | 3 | 13 | 0.014558553 |
| GO:0003854 | 3-beta-hydroxy-delta5-steroid dehydrogenase activity | 2 | 6 | 0.022700748 |
| GO:0016229 | steroid dehydrogenase activity | 2 | 6 | 0.022700748 |
| GO:0033764 | steroid dehydrogenase activity, acting on the CH-OH group of donors, NAD or NADP as acceptor | 2 | 6 | 0.022700748 |
| GO:0004571 | mannosyl-oligosaccharide 1,2-alpha-mannosidase activity | 3 | 17 | 0.030646921 |
| GO:0015924 | mannosyl-oligosaccharide mannosidase activity | 3 | 17 | 0.030646921 |
| GO:0030145 | manganese ion binding | 3 | 17 | 0.030646921 |
| GO:0003873 | 6-phosphofructo-2-kinase activity | 2 | 7 | 0.030923812 |
| GO:0005432 | calcium:sodium antiporter activity | 2 | 7 | 0.030923812 |
| GO:0015368 | calcium:cation antiporter activity | 2 | 7 | 0.030923812 |
| GO:0005539 | glycosaminoglycan binding | 4 | 30 | 0.033301282 |
| GO:0004857 | enzyme inhibitor activity | 12 | 161 | 0.034232015 |

GO enrichment of target genes of DElncRNAs in skin (top 20)

| GO ID | Description | out (258) | All (19473) | pvalue |
| --- | --- | --- | --- | --- |
| GO:0005125 | cytokine activity | 6 | 70 | 0.000329247 |
| GO:0008440 | inositol-1,4,5-trisphosphate 3-kinase activity | 2 | 9 | 0.005920169 |
| GO:0004198 | calcium-dependent cysteine-type endopeptidase activity | 3 | 30 | 0.007166285 |
| GO:0051766 | inositol trisphosphate kinase activity | 2 | 11 | 0.008888107 |
| GO:0005128 | erythropoietin receptor binding | 1 | 1 | 0.013249114 |
| GO:0008455 | alpha-1,6-mannosylglycoprotein 2-beta-N-acetylglucosaminyltransferase activity | 1 | 1 | 0.013249114 |
| GO:0016759 | cellulose synthase activity | 2 | 14 | 0.014326551 |
| GO:0035438 | cyclic-di-GMP binding | 2 | 14 | 0.014326551 |
| GO:0015491 | cation:cation antiporter activity | 7 | 194 | 0.015040971 |
| GO:0008234 | cysteine-type peptidase activity | 5 | 110 | 0.015599878 |
| GO:0016758 | transferase activity, transferring hexosyl groups | 5 | 112 | 0.016742739 |
| GO:0008536 | Ran GTPase binding | 2 | 16 | 0.018566849 |
| GO:0016853 | isomerase activity | 11 | 405 | 0.019654257 |
| GO:0004725 | protein tyrosine phosphatase activity | 5 | 117 | 0.019839566 |
| GO:0046527 | glucosyltransferase activity | 2 | 17 | 0.020860779 |
| GO:0015298 | solute:cation antiporter activity | 7 | 209 | 0.021667474 |
| GO:0008308 | voltage-gated anion channel activity | 4 | 81 | 0.022628353 |
| GO:0003989 | acetyl-CoA carboxylase activity | 2 | 18 | 0.02326601 |
| GO:0016421 | CoA carboxylase activity | 2 | 18 | 0.02326601 |
| GO:0008237 | metallopeptidase activity | 8 | 262 | 0.023900616 |

GO enrichment of target genes of DElncRNAs co-exist in gill and skin (top 20)

| GO ID | Description | out (186) | All (19473) | pvalue |
| --- | --- | --- | --- | --- |
| GO:0004540 | ribonuclease activity | 3 | 21 | 0.001004678 |
| GO:0004714 | transmembrane receptor protein tyrosine kinase activity | 4 | 50 | 0.001317142 |
| GO:0005021 | vascular endothelial growth factor-activated receptor activity | 3 | 25 | 0.00168935 |
| GO:0015491 | cation:cation antiporter activity | 7 | 194 | 0.002666229 |
| GO:0019199 | transmembrane receptor protein kinase activity | 4 | 67 | 0.003866155 |
| GO:0015298 | solute:cation antiporter activity | 7 | 209 | 0.004019853 |
| GO:0005125 | cytokine activity | 4 | 70 | 0.004523762 |
| GO:0003824 | catalytic activity | 88 | 7386 | 0.00542586 |
| GO:0051536 | iron-sulfur cluster binding | 3 | 44 | 0.008520117 |
| GO:0051540 | metal cluster binding | 3 | 44 | 0.008520117 |
| GO:0005451 | monovalent cation:proton antiporter activity | 6 | 187 | 0.009255117 |
| GO:0015385 | sodium:proton antiporter activity | 6 | 187 | 0.009255117 |
| GO:0005128 | erythropoietin receptor binding | 1 | 1 | 0.009551687 |
| GO:0003989 | acetyl-CoA carboxylase activity | 2 | 18 | 0.012556495 |
| GO:0016421 | CoA carboxylase activity | 2 | 18 | 0.012556495 |
| GO:0015299 | solute:proton antiporter activity | 6 | 202 | 0.013173944 |
| GO:0016885 | ligase activity, forming carbon-carbon bonds | 2 | 21 | 0.016914898 |
| GO:0000175 | 3'-5'-exoribonuclease activity | 1 | 2 | 0.019012625 |
| GO:0004532 | exoribonuclease activity | 1 | 2 | 0.019012625 |
| GO:0005415 | nucleoside:sodium symporter activity | 1 | 2 | 0.019012625 |
